# Modeling the Triggering of Saccades, Microsaccades, and Saccadic Intrusions

**DOI:** 10.3389/fneur.2018.00346

**Published:** 2018-05-28

**Authors:** Jorge Otero-Millan, Lance M. Optican, Stephen L. Macknik, Susana Martinez-Conde

**Affiliations:** ^1^Department of Neurology, Johns Hopkins University, Baltimore, MD, United States; ^2^Laboratory of Sensorimotor Research, National Eye Institute, National Institutes of Health, Department of Health and Human Services, Bethesda, MD, United States; ^3^Department of Ophthalmology, SUNY Downstate Medical Center, Brooklyn, NY, United States; ^4^Department of Neurology, SUNY Downstate Medical Center, Brooklyn, NY, United States; ^5^Department of Physiology and Pharmacology, SUNY Downstate Medical Center, Brooklyn, NY, United States

**Keywords:** parkinsonian, progressive supranuclear palsy, square-wave jerk, saccade generation, models, theoretical

## Abstract

When we explore a static visual scene, our eyes move in a sequence of fast eye movements called saccades, which are separated by fixation periods of relative eye stability. Based on uncertain sensory and cognitive inputs, the oculomotor system must decide, at every moment, whether to initiate a saccade or to remain in the fixation state. Even when we attempt to maintain our gaze on a small spot, small saccades, called microsaccades, intrude on fixation once or twice per second. Because microsaccades occur at the boundary of the decision to maintain fixation versus starting a saccade, they offer a unique opportunity to study the mechanisms that control saccadic triggering. Abnormal saccadic intrusions can occur during attempted fixation in patients with neurodegenerative disorders. We have implemented a model of the triggering mechanism of saccades, based on known anatomy and physiology, that successfully simulates the generation of saccades of any size—including microsaccades in healthy observers, and the saccadic intrusions that interrupt attempted fixation in parkinsonian patients. The model suggests that noisy neuronal activity in the superior colliculus controls the state of a mutually inhibitory network in the brain stem formed by burst neurons and omnipause neurons. When the neuronal activity is centered at the rostral pole, the system remains at a state of fixation. When activity is perturbed away from this center, a saccade is triggered. This perturbation can be produced either by the intent to move one’s gaze or by random fluctuations in activity.

## Introduction

Eye motion during exploration of a visual scene consists of a sequence of fast eye movements called saccades, which happen about three times per second, interleaved with fixation periods of relative stability (1, 2). Saccades bring objects of interest toward the high-resolution area of the retina, but have a cost which is not just energetic but also perceptual: we lose visual sensitivity briefly when saccades occur (3). The oculomotor system must decide, at any given moment, whether to initiate a saccade or to remain in the fixation state. This decision involves uncertainty, given the inherent noise in the underlying neuronal signals and cognitive processes.

If we attempt to fixate on a spot, small eye movements, called microsaccades, shift eye position one or two times per second, usually by less than 1° (4). Microsaccades share many characteristics with larger saccades and are likely generated by the same neural system (5, 6). Because microsaccades occur at the boundary of the decision to maintain fixation versus to initiate a saccade, they offer a unique opportunity to study the mechanisms that control saccadic triggering. They also show that, even when we strive to keep our eyes still, the oculomotor system will nevertheless decide, from time to time, to start a saccade (7).

Many models of the saccade generation circuit have been proposed, but they have been rarely tested with small saccades of the size of microsaccades. Indeed, many such models (8) are designed to *not* trigger saccades smaller than a given value (i.e., 2°). In addition, few studies have tested the effect of noise on the system (9–11). This is a critical limitation, in that some models would trigger saccades continuously if noise were added to their inputs. Here, we will implement a model of the saccade triggering system that can initiate both large and small (<1°) saccades and can do so in the presence of noise. We will moreover show that this noise leads to the production of spontaneous microsaccades during fixation.

**Figure 1:**
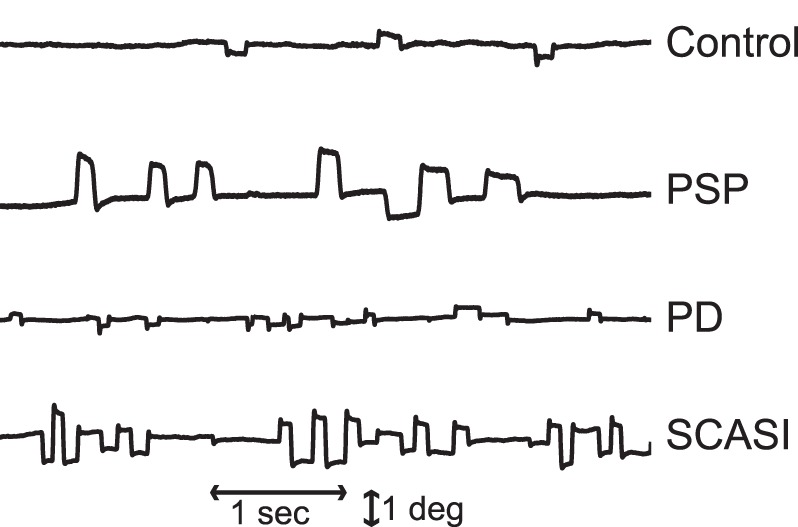
Example of microsaccades and saccadic intrusions during attempted fixation in control subjects and patients with parkinsonian disorders: progressive supranuclear palsy (PSP), Parkinson’s disease (PD), spinocerebellar ataxia with saccadic intrusions (SCASI). Figure adapted from Otero-Millan et al. (12).

Patients affected with neurological disorders can present with abnormal fixational eye movements (1). Thus, the proposed model will also explore the possible mechanisms for the generation of abnormal fixational eye movements, specifically saccadic intrusions. Saccadic intrusions, such as square-wave jerks (SWJs), macro saccadic oscillations, flutter, and opsoclonus, are common in certain neurodegenerative diseases (Figure 1), including progressive supranuclear palsy (PSP), spinocerebellar ataxias, Parkinson’s disease (PD), and others (12, 13).

The model proposed here does not consider other types of eye movements that may affect eye position during fixation, such as drift, tremor, vestibular–ocular reflex (including quick-phases), smooth pursuit, or vergence. Also, we simplify and combine all the possible sources of noise present in the saccade triggering system. These may include inherent neural noise, sensory noise from the visual system, noise from multisensory integration processes, and/or or noise from high-level cognitive processes known to affect eye movements (14).

### Triggers

**Figure 2:**
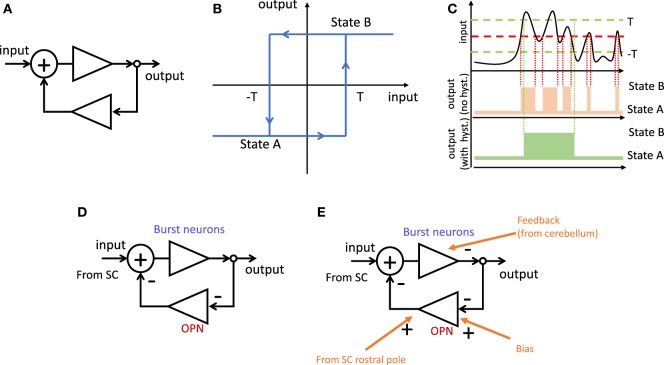
Schmitt trigger. **(A)** Positive feedback loop. **(B)** Hysteresis function corresponding to **(A)**. When the system is in state A, the input needs to reach the value T to switch to state B. When in state B, the input needs to reach −T to switch to state A. **(C)** Effect of hysteresis. Top, example of fluctuating input. Middle, changes of state for a simple threshold (red). Bottom, unwanted switches (in middle panel) can be reduced by having hysteresis with two thresholds (green). **(D)** The same positive feedback loop as in **(A)** formed by double inhibition through brain stem neurons. Because burst neurons (BNs) inhibit omnipause neuron (OPN) which in turn inhibit BNs, the result is net positive feedback loop. **(E)** Further inputs can modulate the behavior of the system. Examples include biasing the threshold or using an additional negative feedback loop to bring the system back to the original state after a switch (that is, ending the saccade).

Many problems in engineering require a robust mechanism to trigger changes between two possible states depending on noisy continuous signals. One common trigger design is known as a Schmitt trigger (15) and relies on a *positive* feedback loop combined with elements with high gain and saturation. This design creates hysteresis, with the useful feature of requiring different thresholds to switch from state A to state B, and from state B to state A. Thus, a minor change in the input after a switch will not produce a switch back to the preceding state (Figure 2).

We propose that a similar mechanism may be used to trigger saccades. A saccade ultimately occurs when the premotor burst neurons (BNs) in the reticular formation start bursting. Another group of neurons in the reticular formation, called omnipause neurons (OPNs), fire at a fairly constant rate between saccades, and stop completely during a saccadic movement (16). These two populations of neurons are linked to each other by inhibitory connections (17). This reciprocal inhibition acts, essentially, as a positive feedback loop. During fixation, the OPNs fire and keep the BNs quiet. Before a saccade, the system switches, so the inhibition of OPNs to BNs decreases while the inhibition from BNs to OPNs increases. This positive feedback ensures that a saccade is initiated quickly and that is not interrupted easily before completion.

The signal that drives the switch between states originates in the superior colliculus (SC). Due to the SC’s topographical organization, saccades of varying sizes and directions can be evoked by microstimulation of different SC locations, with caudal areas triggering large saccades, and rostral areas triggering small saccades (18). Consistent with these microstimulation findings, neuronal activity recordings in the intermediate layers of the SC established that caudal neurons fire before large saccades, and that rostral neurons fire before small saccades (19, 20). Furthermore, neurons in the SC rostral pole are active during fixation but stop during saccades (19). These combined results led to the hypothesis of two SC neuronal populations, with two distinct functions: saccade neurons and fixation neurons. More recent studies challenged this dichotomy though, by showing that rostral pole neurons fire for small microsaccades in an equivalent fashion as more caudal neurons do for larger saccades (6, 21, 22).

The SC projects to both OPNs and BNs, with stronger projections to the OPN from the SC rostral area and to the BNs from the SC caudal area (23, 24). SC projections to the BNs transform the topological coding of saccade magnitude into a rate coding, with the result that BNs fire more for larger saccades.

Here, we implement a model of the trigger mechanism formed by the SC, OPNs, and BNs, to explain how microsaccades may be triggered in the presence of both noise and a constant command to maintain a fixed eye position. We include this trigger in a distributed model of the oculomotor system, and we simulate microsaccadic generation during attempted fixation. We also reproduce some of the experimental observations from prior inactivation or stimulation experiments. Finally, we offer some hypotheses regarding the mechanism that causes abnormal saccadic intrusions in certain neurological disorders.

## Implementation of the Model

The input to the model is the desired gaze location (or location of the target), which is constant in the case of maintained fixation. This command gets updated with every eye movement after a visual feedback delay of 50 ms, to calculate the desired eye position in retinal coordinates. The frontal eye fields (FEFs) send a constant excitatory command to the SC at the corresponding location of the target, and the basal ganglia (BG) send a complementary inhibitory projection, which inhibits the entire SC map except for the location of the target. We also include a noise input to the SC, responsible for the random fluctuations that eventually trigger microsaccades during fixation. This noise input aims to represent the combination of all the potential sources of noise to the SC. The SC combines the different inputs in a map with short-range excitatory connections and long-range inhibitory connections. The SC sends the left and right motor commands to the BNs in the brain stem, as well as a signal to the OPNs, representing mostly the rostral activity. The cerebellum receives the SC command and a copy of the output of the BNs and creates a signal that feeds back to the SC and BNs to stop saccades.

The model builds on both recent and classical results on microsaccade and saccade generation. Hafed and colleagues showed that the activity in the rostral pole of the SC is related to microsaccade generation; thus the SC map can be seen as a continuum of neurons encoding the location of the intended target (22). Shinoda and colleagues showed that the inhibitory burst neurons (IBNs in the pontomedullary reticular formation) send a direct inhibitory projection to the OPNs (17), which is presumably responsible for the lack of OPN activity during saccades. The idea that IBNs inhibit OPNs is supported by results from multiple anatomical and physiological studies (25–27). Van Horn and Cullen showed that OPNs also stop during microsaccades (28), and Van Gisbergen and Robinson found previously that BNs fire for microsaccades as they do for saccades (29).

**Figure 3:**
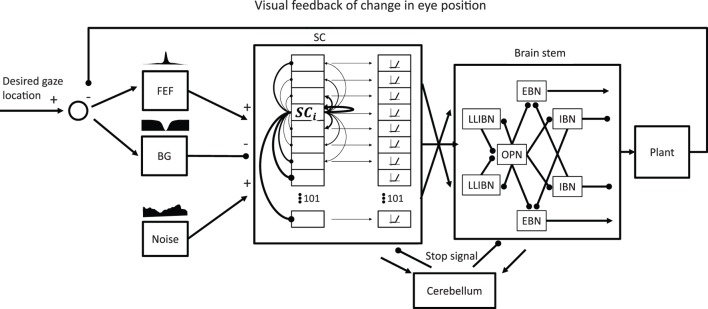
General model structure. The focus of the model is on the network formed by the superior colliculus (SC) and the omnipause neurons (OPNs) and burst neurons (BNs) in the brain stem and how they generate microsaccades in the presence of noise. Thus, the SC and the brain stem reticular formation are the only elements modeled as a network of neurons. All the other elements are “lumped” elements that just attempt to simulate a certain transfer function. The SC receives two complementary inputs indicating the desired gaze location in retinotopic coordinates, one excitatory from frontal eye field (FEF) and one inhibitory from basal ganglia (BG). It also receives a noise input that represents all the possible sources of noise the SC may receive. The SC is a network of 101 interconnected neurons with short-range excitatory connections and long-range inhibitory connections. The output of the SC that drives the BNs includes a nonlinearity that gives more weight to neurons firing more. The SC also drives the OPNs, especially when activity is centered around the center of the SC (representing the rostral pole). The model also includes a simple model of a cerebellar circuit that would produce a stop signal to reset both the SC and the BNs after a saccade. The output of the brain stem drives the eye plant that accounts for the mechanical properties of the eye. The resulting changes in eye position are fed back to the input of the model with a delay of 50 ms.

Figure 3 shows the general structure of the model with its main components. Because we aim to simulate a sequence of eye movements during attempted fixation, and not just an individual saccade, the model must close the visual loop and incorporate the effect of past eye movements. The model includes areas in the cortex, the brain stem, and the cerebellum to control saccade generation. The implementation of each participating area is described in the following sections. Finally, the model includes a final common pathway that creates the pulse-slide-step activity characteristic of the motor neurons and a motor plant that simulates the physical properties of the eye globe.

The model was implemented using Matlab-Simulink (MathWorks Natick, MA, USA) with a 1-ms fixed step for the simulations and the ode3 Bogacki–Shampine solver. Table 1 provides the values for the parameters of the model. To optimize the parameters of the model we first tuned them to produce accurate saccades in the absence of noise. Then, we optimized the parameters of the noise and the parameters relevant to the trigger [connections between OPNs and long lead IBNs (LLIBNs)] to produce microsaccades with comparable properties to real recordings of fixational eye movements. Finally, to simulate the different neurological disorders we only modified one or two parameters at a time to produce saccadic intrusions.

**Table 1:** Model parameters.

Model parameters	
**Burst neurons (BNs)**	
τ_BN_	0.001 s
*b*	8
*B_m_*	800 spikes/s
**Superior colliculus (SC)**	
*F*	500
*A*	3°
*B*	1.4 mm
*A_w_*	1
*C*	1
σ_SC_	0.5 mm
τ_SC_	0.005 s
*S*	5 mm
β*_u_*	0.1
**Frontal eye fields (FEFs) and basal ganglia (BG)**	
σ_FEF_	0.5 mm
σ_BG_	1 mm
**Noise**	
τ_noise_	0.02 s
σ_noise_	0.2 mm
**Brain stem**	
*w*_OPN_LLIBN_	0.0015
*w*_OPN_BN_	0.2
*w*_LLIBN_OPN_	10
*W*_IBN_BN_	0.1
OPN bias	50 spikes/s
**Cerebellum**	
*T*_cblm1_	0.02 s
*T*_cblm2_	0.02 s
*F*_1_	0.1
*F*_2_	0.03
*F*_3_	1
*F*_4_	0.13

### Brain Stem Reticular Formation

The reticular formation circuit we simulate is formed by one OPN, and three BNs on each side: LLIBNs, medium lead excitatory BNs (MLEBNs), and medium lead IBNs (MLIBNs). The OPN inhibits all six BNs. The IBNs inhibit the OPN and the three contralateral BNs. These inhibitory connections serve two distinct roles. First, they ensure that the BNs on only one side are active at the same time (crossing connections from IBNs to all BNs). Second, they control the switching between saccade and fixation states. In between saccades, the OPN inhibits all BNs; during saccades, the IBNs inhibit the OPN.

The connections between the OPN and the IBNs are critical for controlling the triggering of saccades. The connection between the OPN and the MLIBNs must be strong to avoid any firing during fixation; the connection between the OPN and the LLIBNs must be weak to allow for slight changes in drive from the SC to the LLBNs to trigger a saccade. At the same time, the connection between the MLIBNs and the OPN must be strong to completely inhibit it during saccades. Our LLIBNs do not show very long lead activity, but a future implementation of the model could achieve this *via* a population of neurons with variable strength of connections, rather than just a single neuron of each type (30). Similar circuits have been simulated previously to study saccades (31–33).

Every brain stem neuron in the network is modeled as a leaky integrator (time constant τ_BN_) with an exponential output response function, modified from Zee and Robinson (34) with parameter *e*_0_ = 0

(1) $$B(x)=B_{m}*\;(1-{\text{e}}^{-(x-e_{0})/b})$$

### Superior Colliculus

We implemented the SC map following previous models (7, 35). In the model, the left and right SC correspond with a single one-dimensional structure that encodes the horizontal retinotopic space with a set of 101 neurons. The mapping between the coordinates of the retinotopic space (*D*) to colliculus space (*d*) is given by the following formula (36):

(2) d(D)=B∗log((D+A)/A)

with *A* and *B* being the parameters that define the amount of retinal magnification and size, respectively.

Each neuron is characterized by a leaky integrator (with a time constant τ_SC_) of the weighted sum of all its inputs. The output or activation (*a*) of each neuron depends on a nonlinear function (with parameters β*_u_* and *F*) of the output of the integrator (*u*) (7):

(3) $$a(u)=F*1/(1+{\text{e}}^{-\beta_{u}u})$$

Each neuron in the map is connected to every other neuron, and the strength and sign of the connectivity depends on the distance between the two neurons dist(*i, j*), which assumes equally spaced neurons along a 10 mm length (−5 to 5 mm). Neurons that are close to each other receive strong mutual excitation, and neurons that are far from each other inhibit each other. This connectivity is modeled by the synaptic weights of the connections between each neuron. The strength of the connection between neurons *i* and *j* in the map is:

(4) $$w_{ij}=(A_{w}+C){\text{e}}^{-{\left(\text{dist}(i,j)\right)}^{2}/2\sigma_{\text{SC}}^{2}}-C$$

The maximum positive strength is defined by *A_w_* and the maximum negative strength by *C*, dist(*i, j*) is the distance in mm between the two neurons, assuming all neurons are equally spaced and σ_SC_ defines the size of the region that receives excitatory inputs. The input to each SC neuron is defined by the sum of the weighted contributions from all other neurons in the map and the signal coming from the cortex. The cortical signal indicates the desired target location by driving a corresponding patch of SC and inhibiting the rest of the map. We also added a noise input, to simulate microsaccade generation (see following section).

Depending on the characteristics of their firing, SC neurons are typically divided into buildup neurons (if they fire long before the saccade) and BNs (if they fire only around the saccade). Here, we considered the neurons described above as buildup neurons, and we added a burst layer, which is just a thresholded version of the buildup layer. This burst layer acts essentially as a nonlinearity in the center of activity calculation that takes place from SC to BNs. Other mechanisms could achieve the same result, that is, that higher firing at a given SC location gives that location more weight. Regardless of its specific implementation, this feature ensures that the trigger does not get activated during the buildup period of large saccades, while allowing small shifts and increases of the center of activity to trigger microsaccades.

**Figure 4:**
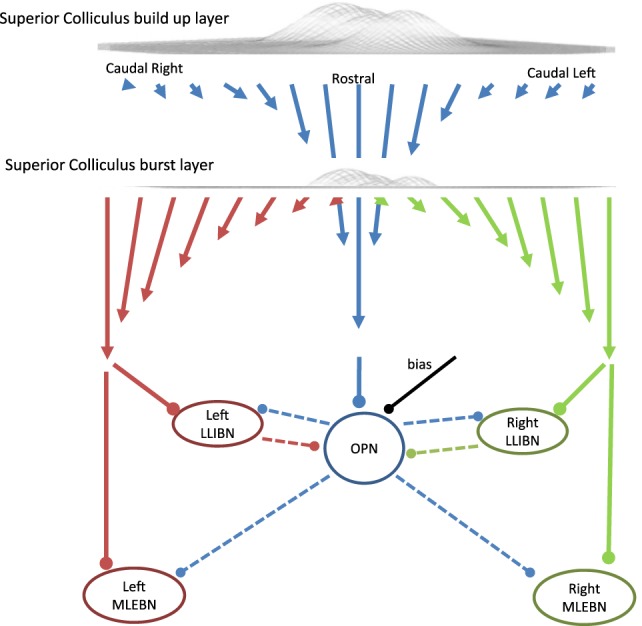
Schematic of the projections from the superior colliculus (SC) to the reticular formation and the triggering circuit. In this model, the SC is a one-dimensional structure with 101 interconnected neurons. During fixation, the activity centers around the middle (which corresponds with the rostral area). The omnipause neuron (OPN) receives excitatory inputs from the buildup layer of the SC with stronger connections (represented as longer arrows) from the more rostral areas. The OPN inhibits all burst neurons (BNs) in the model. The BNs receive excitatory inputs from the contralateral burst layer of the SC with stronger connections from the more caudal areas so the total average input is proportional to the eccentricity of the center of mass of the SC activity. Arrows represent excitatory connections and dots represent inhibitory connections.

The SC sends three projections down to the reticular formation: one to the OPNs, one to the left BNs, and one to the right BNs (Figure 4). The OPNs receive a projection from the entire SC buildup layer, which is stronger from the rostral areas (23). Specifically, the strength of the projection decreases linearly with distance from the rostral end of each SC until it reaches zero at the extremes. That is, the more caudal the SC projection area, the weaker the connection to OPNs. The projection to contralateral BNs comes from the burst layer and is stronger from caudal than from rostral neurons. The strength of the projections from the SC map to the OPN or the BNs is given by:

(5) $$w_{\text{SCOPN}}(i)=1-\left|\text{dist}(i,\;0)\right|/S$$

(6) $$w_{\text{SCBN}}(i)=\text{dist}(i,\;0)$$

where dist(*i*, 0) is the distance in mm between neuron *i* and the neuron at the rostral pole encoding 0° eccentricity and *S* is the size of each SC in mm.

To make the drive from SC to BNs only dependent on the location (rather than the amount) of activity in the map, it is necessary to include a normalization mechanism that implements the center of activity calculation. In our model, we divide the total weighted drive from SC to BNs by the total activity of the SC burst layer. For stability purposes, a small fraction of the buildup activity is also included in the denominator.

### FEF and BG

We have implemented a very simplified version of the outputs of the FEF and the BG that provide the drive and the inhibition that control the activity of the SC. Both outputs present Gaussian profiles (with SDs σ_FEF_ and σ_BG_) centered at the location of the desired target. The level of activity of the FEF also depends on the eccentricity of the target. This simulates the decreased likelihood of a saccade triggered for small retinal errors (37, 38).

### Random Fluctuations

To simulate the eye movements that occur during attempted fixation, we assume that the voluntary command from the cortex to the SC is constant and creates a Gaussian hill of activity centered at the location of the target. To produce microsaccades, we have introduced a noise term to the input to the colliculus. Many sources of neural noise can play a role in microsaccade generation, but here, we lump the effects of all of them into SC activity fluctuations. We have implemented a noise generator that produces noise with temporal and spatial correlations across the SC map. The temporal correlation is created by filtering white noise through leaky integrators of time constant τ_noise_. The spatial correlation is implemented by creating one independent noise source for each neuron and combining them with weights that depend on the distance between neurons, according to a Gaussian function with an SD of σ_noise_. To avoid the triggering of staircase saccades due to persistent noise at one location, the noise pattern is reset after each saccade. Unfortunately, no studies to date have conducted simultaneous recordings of SC neuronal populations, and so there is no good source for the estimation of the parameters of this noise component. We have used a set of values that produces realistic microsaccade distributions.

### Cerebellum

We implemented a very simplified model of the cerebellum that tries to emulate the activity of the fastigial oculomotor region (FOR) related to saccades. FOR is a major cerebellar output nucleus that projects to the brain stem and is involved in saccade generation. Firing of the FOR around saccades is characterized by an early burst in the contralateral side and a late burst in the ipsilateral side (39). Inactivation of the FOR causes changes in saccadic magnitude, with saccades becoming larger or smaller than normal depending on the inactivated side (40). It has been hypothesized that the main role of the FOR could be to track the movement of the eye and send a precise command to stop the saccade on target. This command would correspond to the late burst in the ipsilateral side (41).

To achieve this behavior, our implementation of the cerebellum receives one input from the SC carrying the location of the center of activity in the activity map, and a second input from the reticular formation carrying an efference copy of the MLEBNs activity, which corresponds closely to the eye’s instantaneous velocity during saccades. Though the cerebellum must integrate this efference copy, it is beyond the scope of this study to discuss the specific integration mechanism. Here, we use a second-order system as this integrator with time constants (*T*_cblm1_, *T*_cblm2_), but many other possible implementations would result in similar behavior. The late burst starts when the integrated efference copy (*e*) surpasses the input from the SC (*c*). The early burst corresponds to the activity coming from the SC until the efference copy reaches that point. Different gains in the four different channels [left/right SC (*c_r_*_/_*_l_*) and left/right efference copy (*e_r_*_/_*_l_*)] can achieve different relative timings of the early and late burst. These gains have been tuned to achieve good saccade accuracy over a range of saccade sizes

(7) $$\text{Late }{\text{burst}}_{l/r}=\text{max}((F_{1}e_{r/l}-F_{2}c_{l/r}),0)$$

(8) $$\text{Early }{\text{burst}}_{l/r}=\text{max}((F_{3}c_{r/l}-F_{4}e_{l/r}),0)$$

The late burst acts on the contralateral brain stem to inhibit the ipsilateral BNs. This signal has been defined as a “choke” signal, because it inhibits the BNs regardless of what input they may still receive from the SC.

The cerebellum also projects heavily to the rostral SC (42). For this reason, we have also incorporated a signal that drives the rostral SC to inhibit the caudal SC at the end of the saccade, to terminate the saccade-related burst and restart the rostral SC activity. The magnitude of this projection corresponds to the sum of the two late bursts from each side.

## Results

### Saccades

**Figure 5:**
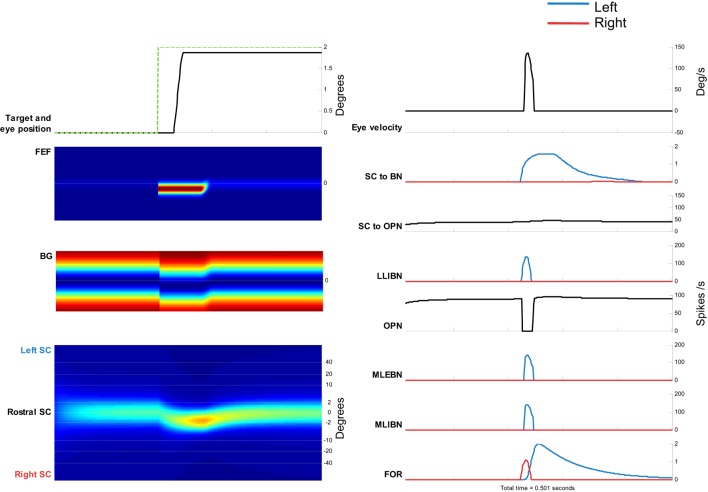
Simulation of a 2° saccade toward the right. Left side, from top to bottom: target movement and simulated eye position. Frontal eye field (FEF) activity. Basal ganglia (BG) activity. Superior colliculus (SC) activity. Color plots show evolution of activity over time in each map. Top, neurons encoding leftward movements. Bottom, neurons encoding rightward movements. Right side, from top to bottom: eye velocity. Total projection from SC to burst neurons (BNs) (left BNs in blue and right BNs in red). Total projection from SC to omnipause neuron (OPN). Firing rate of long lead IBN (LLIBN), OPN, medium lead excitatory BN (MLEBN), medium lead IBN (MLIBN), and fastigial oculomotor region (FOR) (cerebellum).

First, we show the results of simulating a 2° saccade toward the right. Figure 5 shows the corresponding activity in the relevant neurons and areas. The saccade starts when the LLIBN activity is enough to completely inhibit the OPN, which in turn disinhibits the EBN.

The top panels show the eye position and the eye velocity around the saccade. The other panels show the activity of the different elements of the model. The activity of the FEF and BG corresponds to the change in target position, and the activity of the SC dynamically changes toward that location. The drive to the BNs increases while the drive to the OPNs remains relatively constant. At some point, the LLIBNs inhibit the OPNs, letting the MLEBNs and MLIBNs fire and drive the saccade. The activity in the ipsilateral FOR inhibits the BNs, terminating the saccade.

### Simulating Microsaccades During Fixation

**Figure 6:**
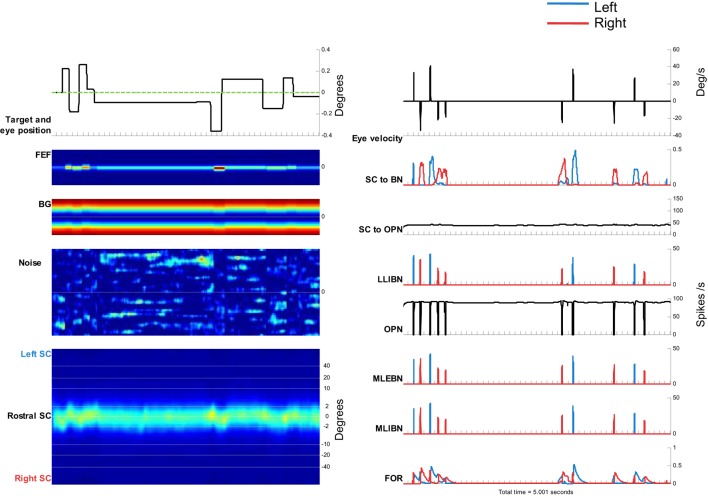
Simulation of microsaccades during 5 s of attempted fixation. Left side, from top to bottom: target movement and simulated eye position. Frontal eye field (FEF) activity. Basal ganglia (BG) activity. Superior colliculus (SC) activity. The color plots show the evolution of activity in each map over time, with neurons encoding leftward movements on the top, and neurons encoding rightward movements on the bottom. Right side, from top to bottom: eye velocity. Total projection from SC to burst neurons (BNs) (left BNs in blue and right BNs in red). Total projection from SC to omnipause neuron (OPN). Firing rate of long lead IBN (LLIBN), OPN, medium lead excitatory BN (MLEBN), medium lead IBN (MLIBN), and fastigial oculomotor region (FOR).

**Figure 7:**
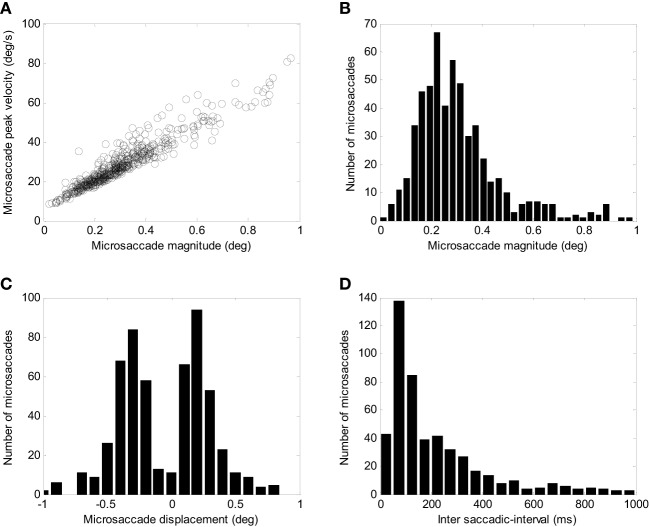
Simulated microsaccade distributions. **(A)** Peak velocity/magnitude relationship. **(B)** Distribution of microsaccade magnitudes. **(C)** Distributions of microsaccade displacements (negative corresponds to amplitude toward the left and positive to amplitude toward the right). **(D)** Distribution of intersaccadic intervals.

To simulate microsaccades produced during attempted fixation, we used a constant target position and added a noise input to the SC. Figure 6 shows the activity in the elements of the model and the resulting eye movement trace for a 5-s simulation. Every time a microsaccade is triggered, the OPN stops firing, in agreement with previous neurophysiological findings (28, 43). Figure 6’s panels show the activity of the different elements of the model, as in Figure 5. This figure also shows the noise introduced to the SC. Figure 7 shows the distributions of various properties of microsaccades collected during a simulation of 100 s of fixation. These distributions are comparable to those obtained from actual fixation conditions (2, 44). We note that, in actual experimental scenarios, fixation periods tend to be much shorter to avoid the subject’s fatigue, which is not a factor in the simulations.

### SC Inactivation

**Figure 8:**
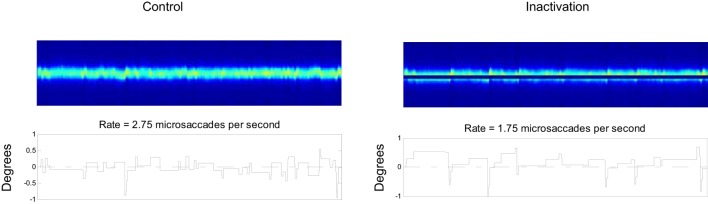
Effects of superior colliculus (SC) inactivation. Left: 20 s of a control simulation. Right: 20 s of a simulation where the output of a set of rostral SC neurons was inactivated. The two top panels indicate the SC activity, and the two bottom panels the corresponding eye positions.

Hafed and colleagues (22) found that inactivating the rostral pole of one colliculus reduces the microsaccade rate. Later, Goffart and colleagues (45) also reported a shift in fixation position following rostral SC inactivation. Here, we asked if our model could simulate those results, by comparing the output of a control setup versus an inactivation setup (Figure 8). We simulated the unilateral inactivation of the rostral SC by nulling the output of the neurons on one side close to the midline (Figure 8). This produced both a decrease in the microsaccadic rate and a shift in the eye position, consistent with the previous empirical results (22, 45). One difference between the present results and previous findings is that our simulations produced an asymmetrical distribution of microsaccade magnitudes, whereas the empirical microsaccade magnitude distributions were reported to be symmetrical. Such difference could be due to some of the simplifications that we have taken in modeling both SC as a single continuous one-dimensional structure.

### Saccadic Intrusions

Saccadic intrusions are saccades that intrude accurate fixation and are prevalent in various neurodegenerative disorders. Here, we simulated the effects of damage to different areas of the oculomotor system to observe the characteristics of the corresponding saccadic intrusions.

**Figure 9:**
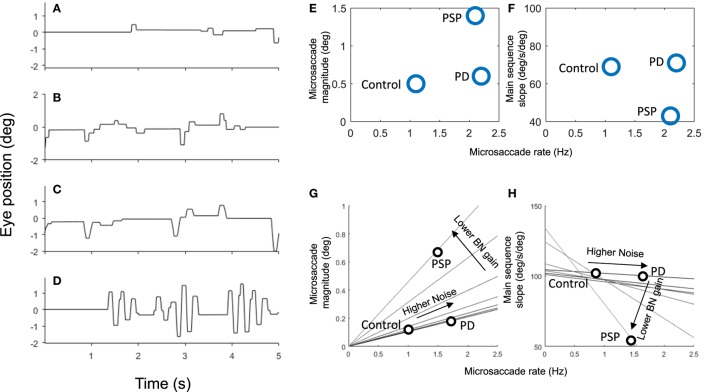
Simulation of 5 s of fixation in **(A)** healthy observers (i.e., using normal parameters), **(B)** Parkinson’s disease (PD), **(C)** progressive supranuclear palsy (PSP), and **(D)** spinocerebellar ataxia. Panels **(E,F)** show the microsaccade magnitude, rate, and velocity (main sequence slope) of three populations of healthy controls, PSP patients, and PD patients from a previous study (12). Panels **(G,H)** show the relationship between microsaccade magnitude, rate, and velocity as we vary two parameters of the simulations. Each line represents a set of simulations with the same burst neuron (BN) gain but varying amounts of noise. As noise increases, the rate (and to some extent the amplitude) of microsaccades increases. Peak velocity is not affected by the noise as long as the BN gain is normal. As we lower the BN gain, the velocity of microsaccades decreases while their amplitude increases. The microsaccade rate also decreases, but in the case of the PSP that is compensated by higher noise.

Figure 9 shows the results of these simulations. First, we simulated the effects of BG impairment by increasing the level of noise in the SC. This resulted in more frequent microsaccades and SWJs, as observed in PD patients (12).

Next, we simulated the increased magnitude and frequency of microsaccades and SWJs in PSP (38), by raising the level of noise and lowering the gain of BNs (parameter *B_m_* in Eq. 1). The decreased gain from BNs is consistent with slower saccades in PSP and may be a consequence of the lack of vertical BNs in PSP. Because BNs (LLIBNs) become less effective on inhibiting OPNs, they require a larger input from SC, which results in increased microsaccade magnitude together with the slower velocity.

Finally, we simulated the effect of cerebellar deficit by decreasing the gain of the stop signal coming from the cerebellum. This produced microsaccades that overshot the target, causing macrosaccadic oscillations typical of some types of spinocerebellar ataxia.

Figures 9E–H show how the simulation replicates the pattern seen in actual patient groups. Here, we model PD by just increasing noise which produces higher saccadic rate, slightly larger saccadic amplitudes, and normal saccadic velocities. We model PSP with both an increase in noise and a decrease on BN gain which produce higher saccadic rates, larger saccadic amplitudes, and lower saccadic velocities.

## Discussion

We have implemented a model based on known anatomy and physiology that successfully simulates the generation of saccades of any size, including the small microsaccades that occur during attempted fixation, and the saccadic intrusions that appear in patients with parkinsonian disorders. The model suggests that noisy activity in the SC map controls the state of a mutually inhibitory network in the brain stem formed by BNs and OPNs. When the activity is centered at the rostral pole, the system stays in a state of fixation. When activity is perturbed away from this center, a saccade is triggered. This perturbation can be produced either by the intent to move one’s gaze or by random fluctuations in activity.

### On the Relationship Between OPNs and SC Rostral Pole

The connectivity between the SC rostral pole and the OPNs has been proven anatomically and physiologically (23, 24). However, it is not clear whether the rostral pole controls the firing of the OPNs (46–48) or merely modulates their behavior. A main argument against the rostral pole controlling OPNs is that, during the gap paradigm, fixation neurons in the rostral pole decrease their activity, though OPN activity remains stable (46–48).

In our model, the rostral pole modulates OPN activity without the need for a one-to-one relationship between the activities of individual SC neurons and OPNs. Because the OPNs receive a projection from a large area of the colliculus (24), it could be that, even if individual neurons of the rostral pole decrease their firing, other neurons that also project to the OPNs increase their firing simultaneously. For instance, one could imagine a situation in which, during the gap period, activity in the rostral pole is lower but wider, keeping a constant drive to the OPNs.

### On the Relationship Between BNs and SC Rostral Pole

Computationally, this projection has been hypothesized to perform a center of activity calculation from the spatial map in the SC to the firing rate of BNs (decomposed into vertical and horizontal components) (49).

For small saccades and microsaccades (less than 1°), the “hill” of activity in the SC may cross over toward the other side. Thus, if the center of activity is calculated only with inputs from neurons from one side, the magnitude of the desired saccade may be overestimated. Moreover, if the activity is perfectly centered at zero, BNs on both sides will receive a non-zero center of activity input. To ensure a zero input to all BNs when activity is centered at zero, there needs to be an inhibitory input to the BNs, to account for neurons encoding small saccades in the opposite direction. Those inputs could come indirectly from the opposite rostral pole as well as from the same one, since there have been reports of neurons within one rostral pole encoding saccades in both directions (6). This issue is irrelevant for larger saccades, where the “hill” of activity is contained within a single side of the colliculus.

### On the Relationship Between BNs and SC Caudal Areas

Here, we set out to answer one fundamental question. Why are microsaccades not triggered by the buildup of activity in the SC caudal areas that precedes a saccade? That buildup must shift the center of activity and thus shift the OPN–BN balance. To solve this problem, we assumed that the projection from SC to BNs is nonlinear, with neurons having a larger effect when they are firing at higher rates. We simulated this by adding a second layer (BN layer) that only fires when the buildup layer reaches a certain threshold (same threshold throughout the map). That way, the brainstem BNs only receive a strong drive when some SC BNs start to fire, so increases in buildup before saccades or due to random fluctuations will not trigger a saccade. Neurons in the SC have been classified in the past as buildup or burst, but this distinction has never been reported in the rostral area. The idea of an SC burst layer is one possible solutions, but there could be other mechanisms for this type of nonlinearity, such as the enhanced synchrony of neurons firing together having a more effective drive on brain stem BNs, which could produce a similar effect. One alternative approach could be to make the connection from rostral SC to OPNs much stronger, so that a saccade is triggered only when the rostral neurons stop firing. However, microsaccades would not be triggered in such case.

### What Happens First?

The nature of the neural event that initiates a saccade has been debated: do saccades start because the OPNs cease to fire, or because the BNs start to fire? Here, we have chosen to consider the system as a whole, since both structures are reciprocally connected (17) and behave as a unit. Thus, we talk about changes in the state of the system, as in going from the fixation state (OPNs fire and BNs silent) to the saccade state (OPNs silent and BNs fire). The positive feedback loop that reciprocally connects both sets of neurons ensures that when activity changes in one neuronal group it also does in the other group.

Thus, the main role of OPNs in this model is to ensure sharp changes between the two states. Without the OPNs, the circuit loses its properties of hysteresis (Figure 1) and any change in SC activity would be reflected directly in the BNs.

### Do Other Signals Bypass the SC to Control Saccade Triggering in the Reticular Formation?

Here, we have given the role of triggering saccades exclusively to the SC and the reticular formation. In our model, all cortical influences on saccade triggering act by affecting SC activity. However, it may be the case that some cortical signals bypass the SC and affect the reticular formation directly. For instance, Valsecchi and Turatto showed that a stimulus that should be invisible to the SC affects microsaccade triggering (50). The supplementary eye fields (SEF) are a potential source for this bypassing signal: the SEF are related to the generation of antisaccades and memory-guided saccades (51), and SEF neurons project directly to the OPN area (52).

## Author Contributions

JO-M and LO designed the model and implemented the simulations. JO-M, LO, SM, and SM-C wrote the manuscript.

## Conflict of Interest Statement

The authors declare that the research was conducted in the absence of any commercial or financial relationships that could be construed as a potential conflict of interest.
